# Goal Pursuit in Organizational Health Interventions: The Role of Team Climate, Outcome Expectancy, and Implementation Intentions

**DOI:** 10.3389/fpsyg.2019.00154

**Published:** 2019-02-05

**Authors:** Anja I. Lehmann, Rebecca Brauchli, Georg F. Bauer

**Affiliations:** Institute of Epidemiology, Biostatistics and Prevention, Public and Organizational Health, University of Zurich, Zurich, Switzerland

**Keywords:** organizational health intervention, context, process, goal pursuit, implementation intentions, healthcare, lean management

## Abstract

In taking a goal pursuit perspective into account, the present study examined associations between the context, process and outcome evaluation of an organizational health intervention (OHI) implemented within 29 teams in a hospital setting. In doing so, team climate for innovation as a context factor was measured at baseline (*N* = 529). Four to six weeks after baseline, *N* = 250 team representatives participated in a 4-day workshop. During the workshop employees formulated collective goals as action plans to be implemented in the nursing wards. Goal pursuit as a process factor was differentiated into (a) a motivational “goal setting” and (b) a volitional “goal striving” phase. The scale of outcome expectancy (measured after the fourth day of the workshop) was used as an indicator for the goal setting phase. For the operationalization of the goal striving phase, action plans were coded with regard to the proportion of formulated implementation intentions (“if-then plans”). After 6 months, the outcome of the intervention was measured on a retrospective impact scale (*N* = 385). The results of the multiple regression analysis and of the multilevel analysis show that both team climate and goal pursuit (outcome expectancy and the proportion of if-then plans) were positively related to the perceived impact of the intervention. Furthermore, the results show that the relationship between team climate and the impact of the intervention was mediated by outcome expectancy. The results highlight the contribution of goal theory within context-process-outcome research that leads to a better understanding of when and why OHIs are effective.

## Introduction

Organizational health interventions (OHIs) aim to tackle organizational issues at their source by changing how work is designed, organized and managed. Research, however, shows inconsistent effects regarding these interventions, due to the complexity of social systems that are difficult to control. This means that some subsystems within the organization addressed by the intervention may support change whereas others may inhibit it (Semmer, 2006). Consequently, it has been argued that a comprehensive analysis of the intervention process and its impact on the intervention outcomes is required to understand when, why and under what circumstances these interventions work (Randall et al., 2005; Semmer, 2006; Biron and Karanika-Murray, 2014). Thus, several researchers have developed frameworks for process evaluation that focus on the implementation of the intervention and how it influences the outcomes (e.g., Nielsen et al., 2010; Biron and Karanika-Murray, 2013; Nielsen and Abildgaard, 2013). Despite the availability of these frameworks, a recent review still claims that lack of theory within process research hinders further progress in intervention evaluation development (Havermans et al., 2016).

With this paper, we aim to take the perspective of goal theory within a specific intervention process stage of an OHI, namely the action planning stage. Generally, an intervention is divided into five stages: initiation/preparation, screening, action planning, implementation, and evaluation (Nielsen et al., 2010, 2013). The action planning stage is a crucial intervention component because it is during this stage that collective goals are formulated, ideally through a participatory approach. This means that employees participate in the development of solutions and formulate goals, according to their work-related needs, to be implemented in the work setting (Nielsen et al., 2010; Abildgaard et al., 2018). It is therefore the assumption that action planning provokes certain behaviors by the employees, namely their engagement in developing and implementing action plans as collective goals, and that these behaviors, rather than the intervention itself, lead to the impact of the intervention (Nielsen and Abildgaard, 2013).

The psychology of action (Lewin et al., 1944, see also Gollwitzer and Bargh, 1996; Gollwitzer and Moskowitz, 1996) offers a theoretical framework for goal pursuit that is applicable to gain a deeper understanding of process mechanisms during the action planning stage. Goals are desired end states that people want to attain (Ryan, 2012). According to the psychology of action (Lewin et al., 1944) goal pursuit is distinguished into two distinct phases: a goal setting and goal striving phase. Goal setting addresses the question of choosing a goal that depends on classical motivational processes. Likewise, goal striving addresses the question of attaining the goal that depends on volitional mechanisms as goal-directed actions and responses. The differentiation between goal setting and goal striving is also prominent within contemporary theories of goal pursuit (see Thürmer, 2013), such as the “Rubicon model” of action phases (Heckhausen and Gollwitzer, 1987; Gollwitzer, 1990).

Although goal pursuit has traditionally been studied at the individual level and within experimental settings, there is current research and acknowledgment that the findings are generalizable for teams working within organizations (Thürmer et al., 2015b). However, if we intend to apply the concept of goal pursuit to OHI research outside the lab, there is a need to consider the context that influences organizational behavior. This means that context should not be regarded solely as a confounder to be controlled, but rather as an important component to understand psychological mechanisms within organizations (Johns, 2001, 2006). Also, within OHI research in particular, there is an emerging acknowledgment of the importance of investigating the intervention context as distinct from the intervention process (Biron et al., 2012; Fridrich et al., 2015). Consequently, some frameworks for OHI evaluation differentiate between context and process in illustrating the influence of context on the outcomes mediated by the intervention process (e.g., Nielsen and Abildgaard, 2013; Nielsen and Randall, 2013; Fridrich et al., 2015).

This study aims to explain the perceived impact of an OHI that was implemented in a hospital setting by team climate for innovation as a *contextual* component and goal pursuit as a *process*-related component. Specifically, we investigate the effect of team climate on the intervention’s impact mediated by goal pursuit, explaining mechanisms leading to the intervention’s effects. This study contributes to the literature in two ways. First, it examines context-process-outcome relations in reacting to the need to understand when, why and under what circumstances interventions work (Randall et al., 2005; Semmer, 2006; Nielsen and Randall, 2013; Biron and Karanika-Murray, 2014). Second, it introduces a goal pursuit perspective as a theoretical framework for the action planning phase. This, in turn, helps to explain context-process-outcome relations more thoroughly.

### Team Climate for Innovation as a Context Factor

Understanding group processes and performance requires careful consideration of the group’s organizational context. Referring to OHIs, context is defined as the underlying frame influencing the implementation and the effects of the intervention (Nielsen and Randall, 2013; Randall, 2013; Fridrich et al., 2015; Ipsen et al., 2015). Team climate, conceptualized as shared perceptions of organizational procedures, practices and policies, refers to a group of people who interact regularly to perform work-related tasks. It can be regarded as a context-related factor as it refers to the immediate social environment in which people create reality to formulate and express perceptions, attitudes and behaviors (Anderson and West, 1998).

Anderson and West developed the team climate inventory (TCI), a team-level concept of how far a team’s values and norms emphasize innovation (West and Anderson, 1996; Anderson and West, 1998). Innovation can be defined as “…the intentional introduction and application within a job, work team, or organization of ideas, processes, products, or procedures, which are new to that job, work team, or organization and which are designed to benefit the job, the work team, or the organization” (West and Farr, 1990, p. 9). For an innovative team climate, four facets have been shown to be important: (1) vision as clearly defined and valued group goals, (2) participatory decision-making, (3) task orientation and (4) support for innovation (Anderson and West, 1998).

The TCI has been validated in several studies internationally (Anderson and West, 1998; Brodbeck and Maier, 2001; Ragazzoni et al., 2002; Mathisen et al., 2004) and has often been applied in the healthcare setting (Jeffcott and Mackenzie, 2008). There is a substantial amount of empirical evidence that has demonstrated the utility of the TCI as a way of examining and predicting healthcare teams’ innovativeness (West and Wallace, 1991; West and Field, 1995; West and Anderson, 1996; Gosling et al., 2003). For instance, a study in an Australian hospital found that effective team work (measured with the TCI) was significantly associated with the effective use of an innovative online evidence system for clinical care (Gosling et al., 2003). In the UK, team climate for innovation predicted team innovation, independently of team size and team tenure, in 27 hospitals (West and Anderson, 1996). Furthermore, teams with shared and clear objectives that emphasized tasks with high quality, who participated in decision-making and were open to innovation, were able to work more effectively and were also more satisfied at their workplace (Poulton and West, 1999; Gil et al., 2005).

### Goal Pursuit as a Process Factor

As already mentioned, goal pursuit is divided into a goal setting and a goal striving phase (Lewin et al., 1944). In this study, outcome expectancy is examined as a goal setting indicator and implementation intentions as a goal striving indicator. These concepts are introduced in detail below.

#### Goal Setting: The Role of Outcome Expectancy

Action planning within OHIs can be understood as a problem-solving activity in which employees select goals that guide decision-making and prospective actions regarding their work-related needs (Nielsen et al., 2010; Abildgaard et al., 2018). Generally, goal setting refers to *motivational* processes (Lewin et al., 1944). Meta-analyses have shown that groups need to set goals. This means that they need to *choose* a desired end state in order to perform well (O’Leary-Kelly et al., 1994; Kleingeld et al., 2011). Thus, action planning toward a goal has particularly a motivational function.

Most goal setting theories (e.g., Atkinson, 1964; Vroom, 1964; Feather, 1982; Ajzen, 1985; Locke and Latham, 1990; Bandura, 1991) have traditionally focused on the formulation of goal intentions that are formed by an expectancy-value paradigm (Gollwitzer, 1990; Brandstätter et al., 2003). This means that the expectations people have explain which kind of goals they choose. People commit to goals in which attainment is perceived as both highly desirable and feasible. Desirability is defined as expectations about the pleasantness of the consequences of goal attainment. Feasibility comprises the beliefs that future actions will be realized (Heckhausen, 1977; Gollwitzer, 1990; Oettingen and Gollwitzer, 2010). Thus, after assessing the desirability and feasibility of an outcome, people commit toward a goal when they believe that the expected value of that outcome is sufficiently high.

Considering the importance of an expected value, outcome expectancies, as beliefs about the consequences of one’s actions, can be considered as a goal-setting indicator. Outcome expectancies are important because people are motivated by a belief that their respective actions (over a longer period) will result in positive outcomes (Ryan, 2012). Expectations of successful outcomes motivate people to put effort into their goals even when obstacles or difficulties arise. Cognitive theories refer to perceived outcome expectancies that have a motivational potential (e.g., Vroom, 1964; Bandura, 1977). Furthermore, outcome expectancies are contagious to others, meaning that people transmit their enthusiasm to other team members who were even not involved in the goal-setting activities, for example, people who did not take part in decision-making workshops in which action plans were formulated (Füllemann et al., 2016). Moreover, it is expected that outcome expectancy is more powerful among groups than among individuals because it refers to the perception of collective agency to generate desired effects (Bandura, 2000). The cohesion of collective expectations indicates an emergent and unified capacity that is more likely than individual efforts to make change possible (Turner, 2005). For intervention outcomes, there is empirical evidence illustrating the positive effects of individual as well as of collective outcome expectancies (e.g., Fridrich et al., 2016; Füllemann et al., 2016).

#### Goal Striving: The Role of Implementation Intentions

Although goal setting includes important motivational components for goal pursuit, it is not always sufficient to reach a goal. Goal striving refers to behavior toward the desired end state (Lewin et al., 1944). This means that individuals aim to realize their intentions by adding goal intentions with volitional components such as plans. Gollwitzer (1999) suggests supporting goals with self-regulatory planning strategies called “implementation intentions” that specify when, where and how action should occur to reach an intended goal. These are so-called “if-then plans” that specify a critical cue or condition *x* (the “if”-part) and pair it with a goal-directed behavioral response *y* (the “then”-part), for example, “If condition *x* is encountered, then I will perform goal-directed response *y*!” (Gollwitzer, 1999). The underlying psychological mechanism refers to the delegation of goal-directed behaviors to environmental stimuli that create a mental representation of the situation and lead to automatic action control (Gollwitzer, 1990; Gollwitzer and Sheeran, 2006).

There is a large body of empirical evidence illustrating the strong effects of implementation intentions for different kinds of goals. A meta-analysis showed medium-to-large effect sizes (*d* = 0.65) of the implementation of intentions to achieve goals compared to goal intention only (Gollwitzer and Sheeran, 2006). These studies were mainly conducted at the individual level and/or in a laboratory setting. Nevertheless, there is also emerging research on the effects of collective implementation intentions (Wieber et al., 2012, 2015a; Thürmer et al., 2015a, 2017) that might also be applied to organizational settings (Thürmer et al., 2015b). Such “we-plans” that specify when, where and how a group acts toward a collective goal should then help collective goal striving that improves performance, and therefore might also be an expedient strategy for reaching goals within OHIs.

For OHI research, the importance of the formulation of action plans that include aims, specific activities, resources and deadlines has been discussed (e.g., Nielsen and Abildgaard, 2013). However, to our knowledge, no empirical study so far has tested these assumptions. Former research on action planning has concentrated on the *content* of these plans rather than on the formulation itself (e.g., Mazzocato et al., 2016; Nielsen and Miraglia, 2017). Although it is undeniable that specific contents can contribute to specific intervention mechanisms, this study shifts the focus to a goal-oriented perspective. This means that action plans formulated as if-then plans illustrate a volitional goal striving indicator that is independent of the content itself.

### Aim of the Study

In taking a goal pursuit perspective into account, this study aims to explore the influence of contextual conditions on the impact of an OHI that is mediated by the intervention process (see Nielsen and Abildgaard, 2013; Nielsen and Randall, 2013; Fridrich et al., 2015). Team climate for innovation is examined as a contextual variable, since one of the hypothesized reasons for attaining collective goals within a change process is their ability to be innovative in sharing an innovative team climate. Furthermore, referring to the psychology of action (Lewin et al., 1944) as a theoretical framework, goal pursuit is examined as a process-related component that is divided into a goal setting phase (that has outcome expectancy as an indicator) and a goal striving phase (that has if-then plans as an indicator).

Considering the need to integrate appropriate multiple level perspectives within OHI research (Martin et al., 2016), we apply a multilevel approach. We consider context and process at the team level, as this intervention refers to collective working environments and shared intervention processes within teams. In particular, team climate is aggregated at the team level as it is conceptualized as a team-level construct (West and Anderson, 1996; Anderson and West, 1998). The process variable outcome expectancy is also aggregated at the team level, as outcome expectancies may be (a) contiguous to other team members through processes of social influence and interaction (Karanika-Murray and Biron, 2013; Füllemann et al., 2016) and (b) expected to be more powerful within collectives (Bandura, 2000; Turner, 2005). The dependent variable is evaluated at both levels as OHIs may have an impact on individuals as well on the collectives sharing the same working environment (Martin et al., 2016).

Based on these considerations we derived the following hypotheses:

*Hypothesis 1*: There is a positive relationship between team climate and (a) the collective impact and (b) the individual impact of the intervention.
*Hypothesis 2*: There is a positive relationship between team climate and goal pursuit: we postulate a positive relationship between team climate and (a) outcome expectancy and (b) if-then plans.
*Hypothesis 3*: There is a positive relationship between outcome expectancy and (a) the collective and (b) the individual impact of the intervention.
*Hypothesis 4*: There is a positive relationship between if-then plans and (a) the collective and (b) the individual impact of the intervention.
*Hypothesis 5*: The positive relationship between team climate and (a) the collective impact and (b) the individual impact of the intervention is mediated by outcome expectancy.
*Hypothesis 6*: The positive relationship between team climate and (a) the collective impact and (b) the individual impact of the intervention is mediated by if-then plans.

## Materials and Methods

### Design

This study was part of an intervention evaluation project that was conducted in a University hospital in Switzerland. The intervention was a participatory lean healthcare intervention that focuses on the improvement of psychosocial health and employee working conditions, like an enhancement of positive affects at work, job resources or the inter-professional collaboration between nurses and doctors. The project lasted from 2013 until the end of 2015.

### Study Sample

This intervention study was conducted within 29 wards (teams). At Time 1, the questionnaire was distributed to *N* = 918 nurses working at different occupational levels. A total of *N* = 529 returned the questionnaire. Among those, *N* = 87 were male *N* = 492 were female. The mean age was 37.47 years old (SD = 11.45). A few weeks after base-line (Time 2), *N* = 250 nurses were chosen by the heads of the departments and the internal project managers to participate in a 4-day workshop in which action plans were formulated each day. The median number of workshop participants was *N* = 8 (Range: 4–22) per ward. 84% were female and the average age was 38 years. After 6 months (Time 3), the questionnaire was distributed to *N* = 945 nurses. Among those, *N* = 385 (with *N* = 120 workshop participants) responded to the follow-up survey. 84% were female and the average age was 39 years. A total of *N* = 171 (*N* = 83 workshop participants) provided data at both Time 1 and Time 3. Of these, 83% were female and the average age was 39 years. This sample is representative to the sample of Time 1, Time 2 and Time 3 based on the demographic data. The dropout rate is similar to comparable research settings (e.g., Ulhassan et al., 2014). Analyses testing for systematic dropout from Time 1 to Time 3 revealed no significant differences between those who only responded at Time 1 to those who responded both times in terms of gender, age and baseline time climate. The study participants generated an anonymous identification code in order to match the surveys from the different time points. Written informed consent was obtained from all participants, but ethical approval was not required according to the local and national guidelines.

### Intervention

The intervention was a lean healthcare intervention with an explicit focus on the improvement of psychosocial working conditions and health. Therefore, a selection of representatives of the respective wards from different occupational fields, including the supervisors, participated in a 4-day workshop. The hospital’s team leaders implemented the four workshop days within a period of 4–6 weeks in a standardized way at each nursing ward. Thereafter, the workshop participants implemented the defined action plans in their wards.

The main goal of the workshops was to identify the best mix of skills and grades per nursing ward in applying *lean principles*. “Lean” refers to a management approach that aims to identify value, map the value stream, create flow and establish pull within and between nursing wards and seeking perfection (Womack and Jones, 1996). Within this intervention context the following *lean strategies* were pursued: (1) improving the productivity/efficiency within ambulatory wards; (2) in stationary wards, investing expensive human resources (high skills/high grades) in highly complex cases; (3) avoiding conflict between quality and efficiency; (4) achieving maximum patient safety through constant monitoring of the performance; (5) implementing safety mechanisms—preventing errors or a stop strategy if errors occurred; (6) assuring a constant flow of materials and patients through the whole hospital (“just-in-time principle”) and reduction of interim storage. This process should optimize the coordination of services.

However, because there is criticism of general lean interventions that may lead to an increase of demands through rationalization, researchers recommend that these interventions be designed with (a) employee involvement and (b) an explicit health-oriented focus (Hasle et al., 2012). Therefore, the workshops followed a participatory approach, which means that employees themselves defined the fields of action and plans to be applied in their own work setting. Furthermore, the workshops included an explicit integration of contents that covered the maintenance and improvement of psychosocial working conditions and employee health.

The workshops took place at the internal training center of the hospital and were led by experts from the field. Furthermore, site visits for so-called gembas (which means “the real place”) were implemented to observe walking distances and waste. During the workshop sessions participants, together with the internal process managers, analyzed the results of the gembas, discussed current and future targeted value-added processes, and (inter-professional) collaboration within the team. Next, participants created several action plans to optimize the value-added processes and the inter-professional collaborations.

In order to simultaneously improve the team climate, job demands and job resources, as well as health among the employees of the nursing wards, workshop participants developed additional action plans concerning these topics during a specially devoted workshop session. They could build on the teams’ baseline results of the employee surveys conducted immediately before the workshop. The action plans were documented electronically into a preconfigured Excel table.

The topics and contents of the 4-day lean workshops implemented in nursing wards are illustrated in Table 1.

**Table 1:** Topics and contents of the 4-day skills-grades-mix workshops implemented in nursing wards.

Topics	Contents
Day 1: Laying the foundations: Analysis of current value stream	Gemba: analysis of current value stream, analysis of process steps, covered distance, identification of general waste. Analysis of interactions between employees, definition of fields of action, formulation of concrete action plans to be implemented
Day 2: Concretion of target process	Presentation and discussion of employee survey results on psychosocial work characteristics, team climate, employee health and work life balance, definition of fields of action, formulation of concrete action plans to be implemented Introduction and planning test run. Introduction to the hospital’s overall lean strategy; lean game. Planning of upcoming implementation of action plans
Day 3: Implementation	Developing target skills-grades profiles specific to each ward. Developing or validating checklists. Evaluating first implementations of action plans. Adapting action plans
Day 4: Implementation and evaluation	Developing detailed target value stream based on developed skills-grades profiles. Quality audits of project and action plans. Site visit of implemented action plans

*Reprinted from Füllemann et al. (2016).*

### Measurements

#### Team Climate for Innovation

At baseline, team climate was measured with the team climate for innovation scale (Van Dick and West, 2013). The scale includes four facets: (1) vision (three items, e.g., “We are in agreement with our objectives”), (2) participative safety (five items, e.g., “In our team there is a feeling of safety and trust”), (3) task orientation (four items, e.g., “We can talk about mistakes openly”), and (4) support for innovation (four items, e.g., “Team members provide practical support for new ideas and their implementation”). The results were measured with a five-point scale (“strongly disagree” to “strongly agree”). In the present study, Cronbach’s Alpha was α = 0.94.

#### Outcome Expectancy

On the fourth day of the workshop the participants responded to a paper-pencil questionnaire that included the outcome expectancy scale. Three items captured expectations about whether the workshops would lead to improvement within the team, in both lean working processes and in working conditions (Fridrich et al., 2016). A sample items is: “Do you think the workshop will have a positive effect on your work?” The replies were rated on a seven-point scale (1 = “no, not at all” to 7 = “yes, very much”). Cronbach’s Alpha was α = 0.84.

#### Implementation Intentions

In total *N* = 878 action plans (*M* = 34, SD = 12 per ward) were formulated within the workshop sessions. In order to generate a quantitative indicator for the implementation intentions, action plans were coded by two independent coders (psychology students at master’s level) regarding an if-then (time point-action) structure was identifiable or not (0 = “no”, 1 = “yes”). Cohen’s Kappa, a measure for inter-rater reliability, was *k* = 0.45 that indicates moderate agreement (Landis and Koch, 1977). As a second step, two researchers (the first author and one psychology student at master’s level) discussed those action plans that had no agreement at the first step (for *N* = 153 cases). This second step was important to handle potential coding biases due to subjectivity and also to reach a unanimous agreement on the total number of if-then plans generated. Finally, *N* = 163 (*M* = 5.56, SD = 3.23 per ward) action plans were identified as plans with an if-then structure. For data analysis, the proportion of if-then plans to the total number of action plans was calculated. The mean ratio of if-then plans per ward was *M* = 0.19 (SD = 0.11).

#### Retrospective Impact Assessment

The perceived impact of the intervention was measured after 6 months using a retrospective impact assessment (RIA) scale that consists of seven items assessing the intervention’s impact from a retrospective viewpoint (Jenny et al., 2015; Fridrich et al., 2016). The scale was measured on a seven-point scale (“not at all” to “yes, definitely”). A sample item is: “Did the intervention project lead to positive outcomes regarding to your work activities?” Cronbach’s Alpha was α = 0.92.

#### Control Variables

Workshop participation was considered as a control variable because former research showed that those employees who participate to a higher extent throughout the intervention process benefit more from the intervention (Landsbergis and Vivona-Vaughan, 1995; Nielsen, 2013; Montano et al., 2014). Furthermore, we considered participation rate (the proportion of employees of a team participating in the workshops) as a group-level control variable. It can be assumed that the greater the proportion is of employees participating within the change process, the more interpersonal influences, diffusion of emotions and change energy within a team will be mobilized (Biron and Karanika-Murray, 2013; Füllemann et al., 2015).

### Data Analysis

First, besides the theoretical considerations to justify data aggregation of individual level data at the team level, we assessed the *ICC(1)*, *ICC(2)* and the mean *r_WG(J)_* for empirical justification for data aggregating. The *ICC(1)* value indicates the proportion of variance accounted for by group membership. A value of 0.01 might be considered as a small effect, of 0.10 as a medium effect and of 0.25 as a large effect (LeBreton and Senter, 2008). The *ICC(2)* value indicates the reliability of the group means. The *r_WG(J)_* is the within-group agreement. For the *ICC(2)* and *r_WG(J)_* it has been suggested that cut-off values should be between 0.60 and 0.70 (Bliese et al., 2002).

To test Hypotheses 1a, 2a, 3a, 4a and 5a we employed multiple regression analyses with all variables at Level 2. To test Hypotheses 1b, 2b, 3b, 4b and 5b we employed multilevel analyses with team-climate, outcome expectancy and if-then plans as Level 2 predictors and RIA as a Level 1 dependent variable. The Level 2 predictors were centered around the grand mean. Within multilevel analysis, we compared several models, starting with the null model that includes only the intercept. In the subsequent steps context predictor variables were included consequently. The improvement of the model can be compared by using the Akaike information criterion (AIC) on a smaller-is-better-basis. The significance level for all analyses was set at *p* < 0.10 in order to guard against type II errors due to the small sample size at Level 2.

To test the mediation Hypotheses 5a, 5b, 6a and 6b three conditions must be met (Baron and Kenny, 1986): (1) the independent variable (team climate for innovation) must be associated to the mediator (outcome expectancy and the proportion of if-then plans), (2) the mediator must be associated to the dependent variable (the perceived impact of the intervention), and (3) a significant relationship between the independent variable (team climate) and the dependent variable (perceived impact of the intervention) will no longer be significant (full mediation) or reduced (partial mediation) when controlling for the mediator (outcome expectancy and the proportion of if-then plans). Additionally, the process macro (Preacher and Hayes, 2008) was applied for the estimation of confidence intervals for the indirect effects among all Level 2 variables (Hypotheses 5a and 6a). The Monte Carlo method recommended by Selig and Preacher (2008) was used to estimate confidence intervals for the hypothesized cross-level 2-2-1 mediation effects (Hypotheses 5b and 6b).

## Results

### Aggregation Analysis and Intercorrelations

Table 2 illustrates the results of the aggregation analysis and the intercorrelations of the variables. The aggregation analysis showed that all *ICC(1)* values were statistically significant and ranged between 0.10 and 0.25. The *ICC(2)* (Range: 0.59–79) and *r_wg(j)_* values (Range: 0.65–0.97) reached the recommended cut-off scores of 0.60–0.70. Thus, it can be concluded that there is sufficient empirical justification for aggregating individual-level variables at the team level.

**Table 2:** Results of the aggregation analysis and the means, SDs and intercorrelations of the variables.

Variables	*M*	*SD*	*ICC(1)*	*ICC(2)*	*r_wg(j)_*	Outcome expectancy	If-then	Participation rate	Workshop participation	RIA (Level 2)
Team climate	3.54	0.32	0.17^∗∗∗^	0.79	0.97	0.329^∗^	−0.007	0.104	–	0.308^†^
Outcome expectancy	5.59	0.61	0.25^∗∗∗^	0.65	0.91	–	0.167	−0.053	–	0.452^∗∗^
If-then	0.19	0.11	–	–	–	–	–	0.031	–	0.356^∗^
Participation rate	0.18	0.06	–	–	–	–	–	–	–	−0.037
Workshop participation	–	–	–	–	–	–	–	–	–	–
RIA (Level 2)	3.42	0.63	0.10^∗∗∗^	0.59	0.66	–	–	–	–	–
RIA (Level 1)	3.42	1.47	–	–	–	–	–	–	0.137^∗∗^	–

*^†^p < 0.10, ^∗^p < 0.05, ^∗∗^p < 0.01, ^∗∗∗^p < 0.001 (one-tailed).*

### Hypothesis Testing

**FIGURE 1:**
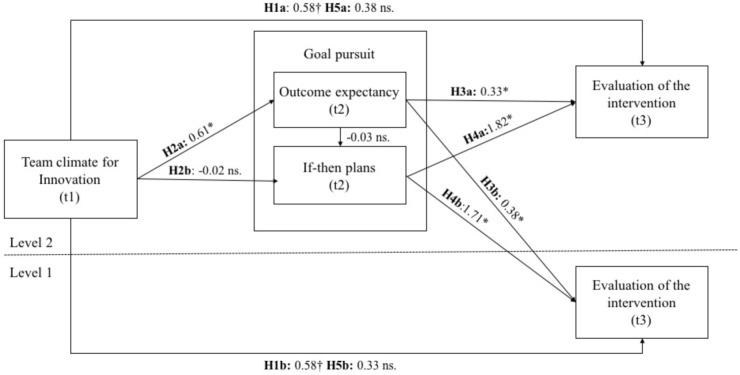
Unstandardized regression coefficients for the relationship between team climate for innovation and the evaluation of the intervention as mediated by goal pursuit (outcome expectancy and if-then plans). H6a and H6b are not illustrated as H2b was not supported by the data. ^†^*p* < 0.10, ^∗^*p* < 0.05 (one-tailed).

Figure 1 illustrates the results of the hypothesized relationships. The detailed analyses of the multiple regression and multilevel analyses are described below.

#### Multiple Regression Analysis

We applied multiple regression analysis to examine the influence of team climate and the collective impact of the intervention that is mediated by goal pursuit (outcome expectancy and the proportion of if-then plans). The results are reported in Table 3. In order to keep the models parsimonious due to the small Level 2 sample size, we did not include participation rate as a control variable since it did not significantly correlate with the outcome based on the correlation analysis (*r* = −0.037).

**Table 3:** Results of the multiple regression analysis.

	Model 1			Model 2			Model 3		
Variable	*B*	SE *B*	Beta	*B*	SE *B*	Beta	*B*	SE *B*	Beta
Const.	1.373	1.25		0.028	1.334		−0.134	1.278	
Team climate	0.584^†^	0.351	0.31^†^	0.341	0.349	0.181	0.382	0.334	0.203
Outcome expectancy				0.394^∗^	0.186	0.392^∗^	0.334^∗^	0.181	0.333^∗^
If-then							1.818^∗^	0.985	0.314^∗^
*R*^2^	0.096			0.233			0.329		
F for change in *R*^2^	2.77^†^			3.807^∗^			3.916^∗^		

*^†^p < 0.10, ^∗^p < 0.05 (one-tailed).*

Hypothesis 1a states that team climate predicts the collective impact of the intervention. This hypothesis was supported by the data (Model 1). The results showed that team climate predicted for the collective impact of the intervention. On a *p*-level *p* < 0.10 (β = 0.310, *p* = 0.054). The second hypothesis is that team climate is positively related with outcome expectancy (Hypothesis 2a), which was confirmed by the data (β = 0.329, *p* = 0.044). However, a positive relationship between team climate and the proportion of if-then plans (Hypothesis 2b) was not supported by the data (β = 0.011, *p* = 0.477). Hypothesis 3a states that outcome expectancy predicts the collective impact of the intervention. Thus, we entered outcome expectancy into the model (Model 2). The results showed that outcome expectancy was a significant predictor for the collective impact of the intervention (β = 0.392, *p* = 0.023), which supported Hypothesis 3a. Hypothesis 4a states that if-then plans predict the collective impact of the intervention, which was also supported by the data (see Model 3; β = 0.314, *p* = 0.039). We also predicted that goal pursuit (outcome expectancy and the proportion of if-then plans) mediates the relationship between team climate and the collective impact of the intervention. In Model 3, the results demonstrated that the direct effect of team climate on the collective impact of the intervention was no longer significant (β = 0.203, *p* = 0.132). The only significant predictors were outcome expectancy and if-then plans. Because there was also a significant relationship between team climate and outcome expectancy, but not with the proportion of if-then plans, we can only conclude a mediation between team climate and the collective impact of the intervention via outcome expectancy (see Baron and Kenny, 1986). The indirect effect was also supported by the bootstrapping approach (Preacher and Hayes, 2008) on a 90%-CI [0.0049; 0.7001].

#### Multilevel Analysis

We applied multilevel analysis to examine the impact of team climate on the individual impact of the intervention that is mediated by goal pursuit (outcome expectancy and if-then plans). Table 4 summarizes the results of Hypotheses 1b–5b. The control variables were included first (Model 1). Model 1 shows that workshop participation was positively related with the individual impact of the intervention (*B* = 0.39, *p* = 0.009). For participation rate, we did not find a significant association with the intervention’s impact (*B* = −0.30, *p* = 0.446). Therefore, as with the multiple regression analysis, we did not include participation rate as a control variable in the following models in order to keep these models parsimonious.

**Table 4:** Results of the multilevel analysis.

	Null model	Model 1	Model 2	Model 3	Model 4
	*B* (SE)	*B* (SE)	*B* (SE)	*B* (SE)	*B* (SE)
Intercept	3.43 (0.12)^∗∗∗^	3.30 (0.13)^∗∗∗^	3.29 (0.12)^∗∗∗^	3.31 (0.11)^∗∗∗^	3.29 (0.11)^∗∗∗^
**Level 1**					
Workshop participation		0.39 (0.16)^∗∗^	0.39 (0.16)^∗∗^	0.38 (0.16)^∗∗^	0.38 (0.16)^∗∗^
**Level 2**					
Participation rate		−0.30 (1.83)			
Team climate			0.58 (0.34)^†^	0.31 (0.33)	0.33 (0.31)
Outcome expectancy				0.42 (0.18)^∗^	0.38 (0.17)^∗^
If-then					1.71 (0.88)^∗^
Variance within groups	1.95 (0.15)^∗∗∗^	1.93 (0.14)^∗∗∗^	1.93 (0.14)^∗∗∗^	1.95 (0.15)^∗∗∗^	1.95 (0.15)^∗∗∗^
Variance between groups	0.22 (0.10)^∗^	0.22 (0.11)^∗^	0.18 (0.10)^†^	0.12 (0.08)	0.09 (0.07)
AIC	1,381.11	1,374.17	1,374.66	1,351.43	1,346.26

*^†^p < 0.10, ^∗^p < 0.05, ^∗∗^p < 0.01, ^∗∗∗^p < 0.001 (one-tailed).*

We then tested Hypothesis 1b that team climate is positively related with the individual impact of the intervention. As shown in Model 2, team climate significantly related to the individual impact of the intervention based on a significance level of *p* < 0.10 (*B* = 0.58, *p* = 0.056). Next, we tested Hypothesis 3b that outcome expectancy is positively related to the individual impact of the intervention. As shown in Model 3, Hypothesis 3b was supported by the data. Outcome expectancy was positively related with the individual impact of the intervention (*B* = 0.43, *p* = 0.019). We then entered the proportion of if-then plans into the model (Model 4), which also supported Hypothesis 4b that the proportion of if-then plans is positively related with the individual impact of the intervention (*B* = 1.71, *p* = 0.035). Compared to all the models, Model 4 had the smallest AIC value and was to be considered as the best-fitting model. Model 4 explained 59% of variance at Level 2. The total explained variance was 6%. Next, we tested Hypothesis 5b that outcome expectancy mediates the relationship between team climate for innovation and the individual impact of the intervention. In Model 4 team climate was no longer significantly related to the individual impact of the intervention, which indicates a mediation between team climate and the intervention’s impact via outcome expectancy (Baron and Kenny, 1986). This indirect effect was supported by the Monte Carlo method (Selig and Preacher, 2008) on a 90%-CI [0.0003; 0.6208]. Hypothesis 6b cannot be supported since there was no association between team climate and the proportion of if-then plans (see Hypothesis 2b in the multiple regression analysis).

## Discussion

This study examined associations between the context, the process and the impact of an OHI by taking perspectives of goal pursuit theory into account. Specifically, the results illustrated that baseline team climate for innovation as a context variable was positively related to the perceived impact of the intervention at team and at individual level (supporting Hypotheses 1a and 1b). Besides team climate as a context factor, this study focused on the process of the intervention in referring to the psychology of action (Lewin et al., 1944) as a theoretical framework for goal pursuit that differentiates between a goal setting and a goal striving phase. The results showed that both outcome expectancy (as a goal setting indicator) and the proportion of if-then plans (as a goal striving indicator) were associated with the perceived impact of the intervention (supporting Hypotheses 3a and 3b and Hypotheses 4a and 4b). Referring to Hypotheses 3a and 3b, this study underlined the importance of positive expectations about forthcoming events and end states as motivational driving forces leading to beneficial intervention effects.

Besides the role of outcome expectancy, the results demonstrated the importance of the way in which action plans are formulated. This implies the critical role of the formulation of action plans as if-then plans explaining the result of effective plans within OHIs. More generally, our findings are in concordance with goal theories (Heckhausen and Gollwitzer, 1987; Gollwitzer, 1990) stating that goal attainment requires following consecutive phases. Goal setting is the first step of goal achievement, whereas goal striving, that includes planning how to get started and achieve a goal, belongs to the next step.

Furthermore, team climate for innovation as a context variable was only associated with outcome expectancy (confirming Hypothesis 2a), but not with the proportion of if-then plans (not confirming Hypothesis 2b). Consequently, and as a result of the mediation analyses, the relationship between team climate for innovation and the impact of the intervention was only mediated by outcome expectancy (confirming Hypotheses 5a and 5b), but not by the proportion of if-then plans (not confirming Hypotheses 6a and 6b). In light of these results, the differentiation between motivational and volitional mechanisms of goal pursuit appears to be important. The non-significant relationship between outcome expectancy and if-then plans further supports the distinctiveness of motivational and volitional states.

Referring to these different mechanisms, this implies that there is no *general* influence of context on process to be concluded since team climate for innovation was only associated with outcome expectancy, but not with if-then plans. Rather, an innovative team climate addresses a *specific* intervention process mechanism, in this case, a motivational mechanism, but not a volitional one. This means that an innovative team climate, expressed by higher expectancies regarding the effects of the intervention, might be able to motivate employees. However, the way that employees formulate action plans as part of a volitional process was not affected by team climate.

The linkage between team climate and motivation can be better understood by taking the mechanisms of social identification into account. Anderson and West (1998) highlight group identification as an important process inducing a shared team climate. Moreover, in referring to perspectives of social identity theory (Tajfel and Turner, 2004; Tajfel, 2010), Ellemers et al. (2004) argue that identification with the team promotes motivation and work engagement which, in turn, fosters identification with collective group goals and group performance. This implies that the underlying mechanism between team climate and motivation is attributable to the degree of social identification with the group.

This study has several theoretical and practical implications. First, this study integrated context and process with outcome evaluation, which is in line with several frameworks that highlight the need to investigate these relationships as underlying mechanisms of change (e.g., Nielsen and Abildgaard, 2013; Nielsen and Randall, 2013; Fridrich et al., 2015). In doing so, this study illustrates beneficial insights for integrating appropriate (psychological) theory for process evaluation. Existing models for the evaluation of implementation processes (e.g., Nielsen et al., 2010; Biron and Karanika-Murray, 2013; Nielsen and Abildgaard, 2013) offer a generic overview of intervention process evaluation. Nevertheless, individual studies are unable to cover all potential process factors and thus some focus on specific intervention stages as, for instance, on the action planning stage is required (Nielsen and Abildgaard, 2013). It is particularly significant to note that during the action planning stage, process evaluation is extremely important to avoid a so-called type III error that occurs when an intervention *per se* is evaluated as ineffective although it was the implementation process that actually went poorly (Biron and Karanika-Murray, 2014). Therefore, a focused view on a specific intervention stage has the advantages that (a) it facilitates applying suitable theory by referring to a specific process component and thus (b) helps to create a deeper understanding of when and why an intervention leads to the desired effects.

Moreover, former research has highlighted the use of qualitative methodology to examine the intervention process as qualitative data leads to a greater understanding of the complexity of employee perceptions, of the intervention development and of its implementation (Nielsen et al., 2006; Randall et al., 2007). Although qualitative data are advantageous to capture multifaceted aspects of the intervention process, it is not possible to empirically test the direct linkages between process and outcomes (Randall et al., 2009). Yet there is a need to integrate the intervention process with outcome evaluation to gain a comprehensive picture of the intervention’s effects (Murta et al., 2007; Egan et al., 2009). Thus, as illustrated in this study, the application of goal theory not only enables the consideration of quantitative measures consistent with theory but also generates an indicator of the types of action plans (as if-then plans) that initially consisted of qualitative information. This, in turn, makes it possible to empirically test the mediating effect of the intervention process.

For practical implications, this study provides support for a motivational step followed by a volitional step in OHIs. Given the importance of team climate for innovation as a motivational context factor triggering high outcome expectancies, it is questionable how such a contextual condition within organizations can be created. Some research on team climate (e.g., Eisenbeiss et al., 2008; Somech and Drach-Zahavy, 2013) refers to social psychological processes for the development of shared norms due to unconscious and reciprocal influences among team members (e.g., Sherif, 1936; MacNeil and Sherif, 1976). These norms should be promoted at the beginning, in the organization’s vision and mission, and continue into the daily routine. Furthermore, it has been shown that specific leadership styles like transformational leadership might foster team climate (Eisenbeiss et al., 2008).

For volitional mechanisms, our findings indicate that forming collective implementation intentions as a means for goal intention within OHIs increases the likelihood that employees will consider the intervention to be successful. Therefore, besides an innovative team climate and high outcome expectancies, a concrete formulation of if-then plans might help teams to ultimately attain their goals.

### Limitations and Future Research

The key strength of this study is the application of goal theory embedded in a context-process-outcome evaluation framework using a multilevel approach in different time points. However, there are some limitations in this research, some of which might be considered for future studies. We have already mentioned the advantages of a tailored analysis focusing on a specific intervention stage, in this case the action-planning stage. It is, however, important to note that we needed to deliberately disregard other intervention stages as well as other context and process factors that may help to explain the underlying mechanisms of an intervention. For instance, team climate does not capture the whole complexity and multifaceted aspects of the intervention context. On may also refer to the organizational climate literature (e.g., Bronkhorst et al., 2015) that further highlights the importance of leadership and supervision. For OHIs in particular, it has been shown that supportive leadership as a contextual variable is associated with the intervention process (Lehmann et al., 2018). Moreover, it would be interesting to examine whether the intervention context (in this case team climate) changes over time. Therefore, future research should focus on other intervention stages considering different theories and/or other context and process factors within different time points that might explain the mechanisms of change.

Second, the calculated number of if-then plans was constructed from qualitative information. Some action plans were formulated in a way that it was not clearly apparent whether they included an if-then structure or not. The remaining scope of interpretation of these sentences might have led to the moderate inter-rater agreement value. To reduce potential subjectivity biases, we discussed all sentences concerning which there was disagreement among the coders. It is, however, to be assumed that there would be fewer ambiguous sentences if the employees were instructed in how to formulate implementation intentions (e.g., to create an experimental condition with an a priori instruction comparable to most research on implementation intentions).

However, although there is also a general claim for methodological rigor within OHI research, experiments are hardly ever applicable within organizations because these are complex settings that cannot be easily controlled (Biron et al., 2008; Nielsen and Miraglia, 2017). Considering the limited applicability of experimental designs, this study follows a so-called realist evaluation approach (Pawson, 2013). Realist evaluation aims to understand when, why and under which conditions intervention work. Therefore, context-process-outcome frameworks can be applied (Nielsen and Miraglia, 2017). This study refers to such a framework and further integrated theoretically driven concepts into it. This, in turn, addresses and relativizes the methodological limitations.

For the if-then plans in particular this means that this study made use of (internal valid) evidence from former experimental research, transferred into the field setting, that reacts to some recent calls to test implementation intentions in the field (Thürmer et al., 2015b). Accordingly, this study represents strengths with regard to external validity because (a) it integrated evidence from former experimental research and (b) the results illustrate an effect of implementation intentions *although* the employees were not aware of how to formulate these sentences (e.g., without an *a priori* instruction). Nevertheless, it is still desirable to replicate these findings in other organizational fields and settings.

Moreover, it would be of interest to understand the underlying mechanisms of implementation intentions in more detail. For instance, behavioral and physiological studies illustrate how the processes of implementation intentions are translated into action (Wieber et al., 2015b). For OHI research, future studies should focus on the understanding how implementation intentions affect collective action within teams. This would give further insights in explaining the effectiveness of collective implementation intentions within OHIs.

Additionally, the small sample size of 29 teams could be regarded as a limitation. However, finding relationships in a small sample illustrates large effect sizes (Cohen, 1992).

Finally, the assessment of the impact of the intervention could be regarded as a limitation since it refers to a retrospective perception. However, sensemaking is a retrospective activity (Weick, 1995) that explains an important mechanism of an OHI (Nielsen and Abildgaard, 2013). Moreover, previous studies have shown that the RIA can be applied as a generic indicator to explain change in intervention outcomes (Jenny et al., 2015). Nevertheless, the results rely on self-reported data. Future studies might consider further performance indicators to validate the results with an objective measure.

## Conclusion

The results of this study highlight the contribution of a goal pursuit perspective within context-process-outcome research that leads to a better understanding of when and why OHIs are effective. Specifically, we found that team climate for innovation as a context-factor mediated by outcome expectancy as a goal-setting indicator predicts the perceived impact of the intervention. Referring to the distinction between goal setting and goal striving, this research further highlighted that OHIs are more effective when, in addition to setting goals, employees also strive to attain their goals by formulating action plans as implementation intentions.

## Author Contributions

The data analysis and manuscript was prepared by AL with support from RB and GB. All authors critically reviewed and contributed to the manuscript and approved the final version.

## Conflict of Interest Statement

The authors declare that the research was conducted in the absence of any commercial or financial relationships that could be construed as a potential conflict of interest.
